# The chaperone system in cancer therapies: Hsp90

**DOI:** 10.1007/s10735-023-10119-8

**Published:** 2023-03-18

**Authors:** Charbel A. Basset, Everly Conway de Macario, Lavinia Giovanna Leone, Alberto J.L. Macario, Angelo Leone

**Affiliations:** 1 Department of Biomedicine, Neuroscience and Advanced Diagnostics, Institute of Human Anatomy and Histology, University of Palermo, Via del Vespro, 127, Palermo, PA 90129 Italy; 2 Department of Microbiology and Immunology, School of Medicine, University of Maryland at Baltimore-Institute of Marine and Environmental Technology (IMET), Baltimore, MD USA; 3 Euro-Mediterranean Institute of Science and Technology (IEMEST), Palermo, Italy; 4 School of Medicine, University of Palermo, Palermo, Italy

**Keywords:** Hsp90, Chaperone system, Molecular chaperone, Chaperonopathies, NF-kB, Akt, Negative chaperonotherapy

## Abstract

The chaperone system (CS) of an organism is composed of molecular chaperones, chaperone co-factors, co-chaperones, and chaperone receptors and interactors. It is present throughout the body but with distinctive features for each cell and tissue type. Previous studies pertaining to the CS of the salivary glands have determined the quantitative and distribution patterns for several members, the chaperones, in normal and diseased glands, focusing on tumors. Chaperones are cytoprotective, but can also be etiopathogenic agents causing diseases, the chaperonopathies. Some chaperones such as Hsp90 potentiate tumor growth, proliferation, and metastasization. Quantitative data available on this chaperone in salivary gland tissue with inflammation, and benign and malignant tumors suggest that assessing tissue Hsp90 levels and distribution patterns is useful for differential diagnosis-prognostication, and patient follow up. This, in turn, will reveal clues for developing specific treatment centered on the chaperone, for instance by inhibiting its pro-carcinogenic functions (negative chaperonotherapy). Here, we review data on the carcinogenic mechanisms of Hsp90 and their inhibitors. Hsp90 is the master regulator of the PI3K-Akt-NF-kB axis that promotes tumor cell proliferation and metastasization. We discuss pathways and interactions involving these molecular complexes in tumorigenesis and review Hsp90 inhibitors that have been tested in search of an efficacious anti-cancer agent. This targeted therapy deserves extensive investigation in view of its theoretical potential and some positive practical results and considering the need of novel treatments for tumors of the salivary glands as well as other tissues.

## Introduction

The Chaperone System (CS) of an organism is composed of molecular chaperones, co-chaperones, chaperone co-factors, and chaperone receptors and interactors (Macario and Conway de Macario 2019, 2020). Molecular chaperones, the chief members of the CS, do not act alone but form teams and these usually interact with other teams forming functional networks. The CS is deeply involved in cellular physiology and, thus, any disruption of this system can lead to pathology. This makes the study of the CS in any disease of crucial importance. Some molecular chaperones are heat shock proteins (Hsps) and they function constitutively and upon cellular stress (Macario 1995; Macario and Conway de Macario 2007; Dahiya and Buchner 2019; Edkins and Boshoff 2021). The involvement of molecular chaperones in disease, including cancer, has been established (Hoter et al. 2018b, 2019; Lang et al. 2019; Macario and Conway de Macario 2021; Johnson 2021). Hsp90 has been the target of many investigations in neoplasms because it plays a pivotal role in chaperoning proteins key to tumorigenesis (Schopf et al. 2017; Hoter et al. 2018b, 2019; Siebert et al. 2019; Birbo et al. 2021). In addition, the importance of Hsp90 in carcinogenesis is apparent by the many efforts to develop inhibitors of this chaperone for use as anticancer agents (Schopf et al. 2017; Birbo et al. 2021; Saha et al. 2021). Hsp90 inhibitors were among the first Hsp inhibitors to be evaluated for use in anti-cancer treatment, and as these first-generation inhibitors were not satisfactory, second-generation compounds were developed, some of which have been selected for clinical trials. In this review, we delve into the molecular mechanisms by which Hsp90 promotes carcinogenesis and outline our current understanding on how it can be targeted for treating cancer, including those of the salivary glands.

## The chaperone system

**Fig. 1:**
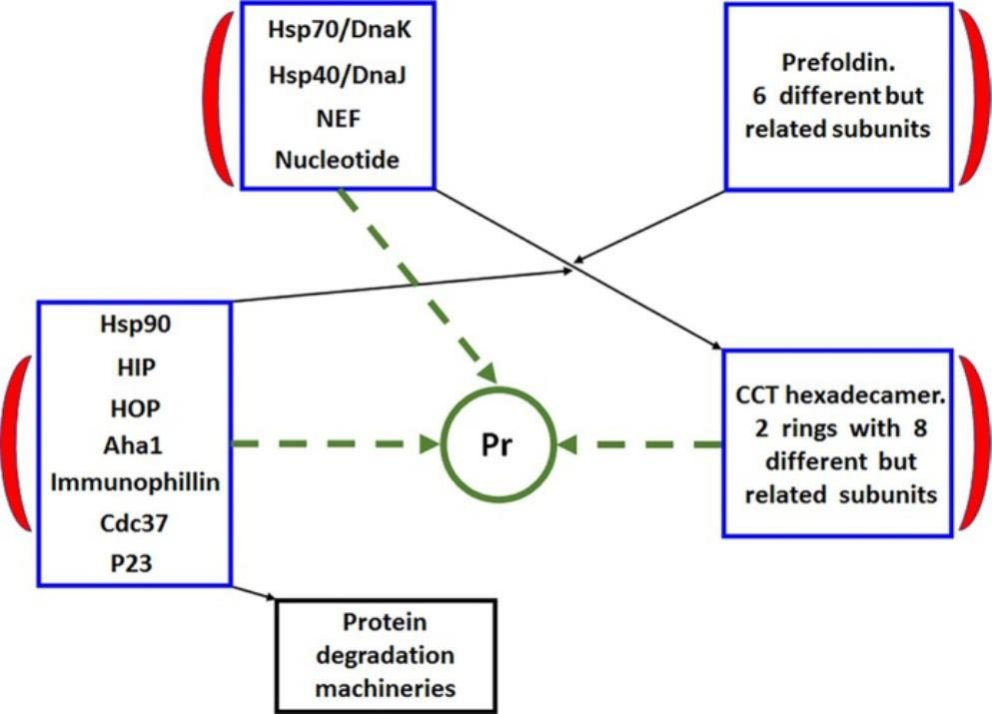
Important components of the human chaperone system that play a role in carcinogenesis forming functional teams and networks. The Hsp70/DnaK, Prefoldin, Hsp90, and CCT teams (framed in blue) interact with one another in various ways forming functional networks to maintain protein homeostasis with the result being the production of fully functional protein molecules (Pr) and the removal of defective peptides (protein degradation machineries, e.g., the ubiquitin-proteasome system, other proteases, and autophagy mechanisms). Each team undergoes functional cycles with turnover and exchange of components as indicated by the red moon-shaped icon. For details see text, Fig. 3, and (Macario and Conway de Macario 2005, 2020; Wandinger et al. 2008; Schopf et al. 2017; Dahiya and Buchner 2019; Biebl and Buchner 2019; Edkins and Boshoff 2021; Knowlton et al. 2021; Birbo et al. 2021; Johnson 2021)

An important concept to bear in mind for understanding the role of molecular chaperones in carcinogenesis is that the molecular chaperones do not act alone but rather are part of functional teams and networks (Macario and Conway de Macario 2019, 2020) as schematically illustrated in Fig. 1, in which the Hsp90 team is shown forming a network with the Hsp70, prefoldin, and CCT (Chaperonin Containing T-CP1) teams. The CS interacts with the ubiquitin-proteasome system (UPS) and with the chaperone-mediated autophagy machinery as part of its canonical functions in maintaining protein homeostasis (Nedelsky et al. 2008; Tekirdag and Cuervo 2018; Kocaturk and Gozuacik 2018; Macario and Conway de Macario 2019; Rios et al. 2021; Liao et al. 2021). The CS has also non-canonical functions exemplified by its involvement in inflammatory processes, autoimmune diseases, and cancer (Henderson et al. 2013; Calderwood and Gong 2016; Jeffery 2018; Saini and Sharma 2018; Macario and Conway de Macario 2019; Milani et al. 2019; Caruso Bavisotto et al. 2020; Macario and Conway de Macario 2021). Abnormal CS components can cause diseases, the chaperonopathies, in which the chaperone is a determinant etiopathogenic factor (Macario and Conway de Macario 2005). Hsp90 chaperonopathies, including abnormalities of its co-chaperones, are involved in various disorders (Johnson 2021). Interestingly, chaperonopathies can also be prominent in aging, a universal and multisystemic process deemed to be the underlying cause of the chronic diseases affecting the elderly. For instance, Hsp90 levels and chaperoning capacity were diminished in the cytosol of hepatocytes of older rats when compared with those of younger rats (Nardai et al. 2002). Chaperonopathies can be genetic (arising from mutation of molecular chaperone genes) or acquired (e.g., those chaperonopathies caused by aberrant post-translational modifications of the chaperone protein). The alterations of the cellular and tissue levels of chaperones are characteristic of quantitative chaperonopathies. If the alteration of the level, or expression, or structure-function of a chaperone is the main etiopathogenic factor, the disease is classified as a primary chaperonopathy, but if the alteration is the consequence of the disease this is classified as a secondary chaperonopathy. Chaperonopathies are further classified based on their biologic and molecular mechanisms into by defect, by excess or by mistake (Macario and Conway de Macario 2007). From the practical standpoint for histopathologists and clinicians, identification of chaperonopathies is bound to improve the accuracy of diagnosis and patient management. Identification and characterization of the specific role of chaperonopathies in the molecular mechanisms of carcinogenesis will open opportunities to develop efficacious therapies targeting specific components of the CS, the basis of chaperonotherapy (Macario and Conway de Macario 2007; Cappello et al. 2014).

## Molecular chaperones

Cells can maintain functional proteins under stress conditions by various mechanisms, including those in which the CS is the main actor. The chief components are the molecular chaperones, many of which are called heat shock protein (Hsp) because their encoding genes are inducible by a sudden temperature elevation or heat shock. Hsps can be divided into two categories: constitutively expressed and inducible, with the former involved mostly in house-keeping functions and the latter coming into action in response to stress (Macario 1995; Stetler et al. 2010; Dahiya and Buchner 2019; Edkins and Boshoff 2021). The CS canonical role is maintenance of protein homeostasis whereas its non-canonical functions are related to immune and inflammatory reactions and carcinogenesis (Hightower et al. 2000; Vitadello et al. 2010; Henderson et al. 2013; Thanos et al. 2014; Calderwood and Gong 2016; Jeffery 2018; Saini and Sharma 2018; Cappello et al. 2019; Macario and Conway de Macario 2019; Milani et al. 2019; Caruso Bavisotto et al. 2020; Macario and Conway de Macario 2021).

Although Hsps acquired their name because their genes are inducible by heat shock (Ritossa 1962; Tissiéres et al. 1974), they can also be expressed in response to an array of stressors such as irradiation; inflammation; heavy metals; antibiotics; alcohols; and oxidative, osmotic, pH, and mechanical trauma (Macario 1995; Jolly and Morimoto 2000; Papp et al. 2003; Dubrez et al. 2019). Molecular chaperones are classified since many years ago into groups on the basis of molecular weight, encompassing the following ranges (in kDa): <34; 35–54; 55–64; 65–80; 81–99; 100–199; and ≥ 200 (Macario 1995; Macario and Conway de Macario 2019); within these groups are families of phylogenetically related Hsps such as the Small Hsp (those with the alpha-crystallin motif), Hsp40/DnaJ, CCT, and Hsp70/DnaK families. In the literature, the nomenclature of molecular chaperones/Hsps is rather confusing and, in an attempt to remedy this disorder, a set of rules has been proposed (Kampinga et al. 2009).

In urothelial, prostate, some types of salivary gland, neuroepithelial, large bowel cancers one or more Hsps are increased in the tumor tissue (Cappello et al. 2003, 2006a; Wang et al. 2013; Rappa et al. 2013, 2014; Campanella et al. 2015; Basset et al. 2020). However, in other types of salivary gland and bronchial epithelial cancers Hsps are decreased (Cappello et al. 2006b; Basset et al. 2021). Neoplastic cells use the cytoprotective function of Hsps to their advantage to survive, grow, spread, and evade the immune response and apoptosis (Hoter et al. 2019; Lang et al. 2019; Edkins and Boshoff 2021; Birbo et al. 2021).

## Hsp90: structure and isoforms

Hsp90 is found in all living kingdoms besides Archaea (Chen et al. 2006). Bacteria harbor one copy of Hsp90 that is dispensable for bacterial life even in the presence of heat stress (Biebl and Buchner 2019). Evolutionarily, gene duplication gave rise to paralogs in eukaryotes reflecting the complexity of multicellular organisms and their increased need of chaperoning a larger number of proteins in contrast with unicellular beings. At least one Hsp90 gene is required for life in eukaryotes, even under normal conditions, which exemplifies the correlation in evolution between increasing complexity of organisms and a parallel increase of complexity of the CS (Biebl and Buchner 2019). Two isoforms are found in yeast, i.e., the constitutive Hsc82 (Hsp90β in human) and the heat-shock inducible isoform Hsp82 (Hsp90α in human), while humans have two extra organelle-specific isoforms: TRAP1 is confined in the mitochondrial matrix and intermembranous space and GRP94 resides in the endoplasmic reticulum (ER) (Hoter et al. 2018b; Biebl and Buchner 2019).

The molecular structure of Hsp90 encompasses three highly conserved domains: N-terminal (NTD), middle (MD), and C-terminal (CTD) domains (Hoter et al. 2018b; Biebl and Buchner 2019). Whilst slight but functional differences are found between orthologs and paralogs, this structure is fundamentally conserved across species (Chen et al. 2006). Eukaryotes possess a charged linker region (CR) that links NTD to MD. The NTD bears the nucleotide-binding motif which is essential for ATP hydrolysis to mediate the Hsp90 cycle and client binding (Panaretou et al. 1998). ATP binds in a unique conformation to the ATP-binding pocket in the NTD, which allows specific inhibition of Hsp90 via competition with ATP binding, and this results in proteasomal protein degradation (these mechanisms will be discussed in more detail in the last Section of this review) (Chiosis et al. 2002; Biebl and Buchner 2019). The CR is a highly charged, dynamic and flexible region that modulates the chaperone function by increasing flexibility and dynamicity of Hsp90 in a crowded space such as that found inside eukaryotic cells with many proteins and other molecules (Shiau et al. 2006; Tsutsumi et al. 2009). Bacterial HtpG and TRAP1 lack the CR (Biebl and Buchner 2019). The MD contains the binding sites for client proteins and co-chaperones. The arginine residue 380 (Arg380) in MD modulates ATP hydrolysis and, thus, the chaperone function and cycle through its direct contact with the γ-phosphate of ATP on the NTD (Meyer et al. 2003). CTD mediates the homodimerization of Hsp90 that results in transient amino-terminal dimerization (Meng et al. 1996). CTD contains a nucleotide-binding domain that allosterically regulates the NTD ATPase activity (Soti et al. 2003). CTD of cytosolic and ER Hsp90 contain MEEVD and KEDPL motifs, respective (Hoter et al. 2018b). MEEVD motif serves as the binding site for co-chaperones with the TPR (tetratricopeptide-containing repeats)-domain (Garg et al. 2016). Hsp90 teams up with different co-chaperones, depending on the function and on the locale in which it functions and, thus, abnormalities in the chaperone itself and on its co-chaperones cause a variety of pathological and clinical disorders (Johnson 2021).

## Hsp90 cycle

The ATPase cycle follows a similar mechanism of structural rearrangements upon nucleotide binding in all Hsp90 isoforms, paralogs and orthologues (Wandinger et al. 2008), but some isoform-specific differences do exist (Biebl and Buchner 2019). The cycle starts with the homodimer Hsp90 being in an open conformation state. The misfolded or nascent polypeptide is bound to Hsp70/Hsp40/ADP complex (Walter and Buchner 2002) that is further stabilized by the docking of HIP (Hsp70-interacting protein) to Hsp70 (Chaudhury et al. 2006), which is followed by the binding of the client protein to Hsp90. The binding is facilitated by the co-chaperone HOP (Hsp90-Hsp70 organizing protein) that mediates the interaction between Hsp70 and Hsp90 (Murphy et al. 2001). At this point of the cycle, the co-chaperones Cdc37 intervenes to complete the loading of the client onto the Hsp90 (Caplan et al. 2007) and immunophilins, such as FKBP51 and FKBP52 (FK506-binding protein 51 and 52, respectively), are added, while Hsp70, Hsp40, HIP, and HOP dissociate, with the result being an activated hetero-oligomeric team (Kosano et al. 1998). ATP binding to the NTD followed by closure of the ATP lid onto the bound ATP yields a first intermediate state. This starts a series of conformational rearrangement that drives the dimerization of the NTDs as they swap segments forming the first closed state (Ali et al. 2006). This event is then followed by a shortening of the distance between the MD and the NTD bringing the two split ATPase sites together, forming the ATPase-competent conformation (second closed state) (Prodromou 2000; Cunningham et al. 2008, 2012; Hessling et al. 2009). Further binding of the co-chaperones p23 and the activator of Hsp90 ATPase homologue (Aha1) to the complex initiates ATP hydrolysis, while the latter additionally enhances the release of still-bound co-chaperones (Ali et al. 2006). The cycle conclusion is marked by release of ADP and amino-terminal dissociation post-hydrolysis.

## Hsp90 regulation

Hsp90 activity is regulated at the transcriptional level by HSF1 (heat shock factor 1), the master regulator of the heat shock response (HSR) (Åkerfelt et al. 2010; Richter et al. 2010). HSF1 is in turn regulated by a negative feedback loop in which Hsp90 and Hsp70 levels are determinant factors to suppress the activity of HSF1 (Zou et al. 1998; Ali et al. 1998; Lackie et al. 2017; Kijima et al. 2018; Kmiecik et al. 2020).

Hsp90 is subject to post-translational modifications (PTMs) including phosphorylation, acetylation, SUMOylation, S-nitrosylation, methylation, and ubiquitylation that regulate its function by impeding ATPase activity, conformational changes, accessibility to binding sites, and interaction with co-chaperones, ultimately resulting in impairment of the chaperone function and client maturation (Schopf et al. 2017; Hoter et al. 2018b; Biebl and Buchner 2019).

The intricacy of the mechanisms of regulation of Hsp90 is further increased by the interplay between co-chaperones and Hsp90 protein complexes that modify the function of some client proteins (Zuehlke and Johnson 2010; Garg et al. 2016; Hoter et al. 2018b; Biebl and Buchner 2019).

## Hsp90 functions

Hsp90 isoforms differ in their functions according to their locale or residence. Cytosolic Hsp90 (Hsp90α/Hsp90β) sits at the center of various cellular processes and biological pathways as a master regulator of cell signaling, cell cycle and differentiation, and cytoskeleton remodeling. GRP94 on the other hand, is involved in: (1) the maturation, folding and assembly of secretory and membrane-bound proteins such as Toll-like receptors (TLRs) and integrins; (2) the binding of calcium in the ER contributing to Ca2 + homeostasis in the organelle and the cell as a whole; (3) acting as a major checkpoint in the ER chaperone network for ER quality control; and (4) mediating with BiP (GRP78) the unfolded protein response (UPR) upon ER stress, while TRAP1 specializes in maintaining mitochondrial integrity by opposing mitochondrial oxidative stress-generated apoptosis. It is now established that chaperones, including Hsp90, are not only intracellular molecules but are also functioning extracellularly. Under various stress and necrotic conditions (Basu et al. 2000; Berwin et al. 2001), all the Hsp90 isoforms except Hsp90β can be secreted outside the cell to participate in immunogenic and inflammatory activities (Song et al. 1995; Chen et al. 1996; Simmons et al. 1999; Jackson 2012; Calderwood et al. 2016). Extracellular Hsp90 displays non-canonical functions, as illustrated by its being expressed on the surface of tumor cells surface (Altmeyer et al. 1996) while chaperoning tumor peptides and presenting them to MHC class I molecules of antigen-presenting cells (APCs), a process known as “cross presentation” (Lammert et al. 1997; Spee and Neefjes 1997; Evdokimovskaya et al. 2012). Hsp90 can also be secreted to participate in wound healing and cell motility (Li et al. 2007, 2012; Cheng et al. 2008; Woodley et al. 2009; Evdokimovskaya et al. 2012; Hoter et al. 2018a).

## Molecular mechanisms of Hsp90 in cancer

The role of Hsp90 in cancer has been thoroughly studied throughout the years due to its specific function pertaining to chaperoning mutated oncogenes as its main targets, favoring tumor growth and progression (Hoter et al. 2018b). Hsp90 mediates cell cycle progression and activates signaling kinases via Cdc37 co-chaperone interactions that are crucial for activating anti-apoptotic and proliferative pathways essential for carcinogenesis (Wang et al. 2019). Neoplastic cells become “chaperone addicted” as their demand for Hsps, including Hsp90, exponentially increases. This becomes a vicious cycle where an increase in mutated proteins drives the induction of Hsps, increase in translational processes and expression of proteins leading to transformation (Ciocca et al. 2013). Pronounced expression of Hsp90 in several cancer types has been reported (Rappa et al. 2014; Kamm et al. 2019; Caruso Bavisotto et al. 2019; Gorska-Ponikowska et al. 2020; Barone et al. 2021). Therefore, Hsp90 has been proposed as a potential candidate for biomarker studies as its increased expression in neoplastic tissue correlated directly with clinically advanced stages and poor prognosis (Pick et al. 2007; Chiu et al. 2011; Chen et al. 2015; A. Ansa-Addo et al. 2016; Maddalena et al. 2017; Feng et al. 2019). However, as reported for other Hsps, Hsp90’s pattern of expression is tissue and cancer-type specific as shown by a decrease in Hsp90 tissue levels in infiltrative lobular carcinoma in contrast with other ductal and lobular breast cancers (Zagouri et al. 2010).

**Fig. 2:**
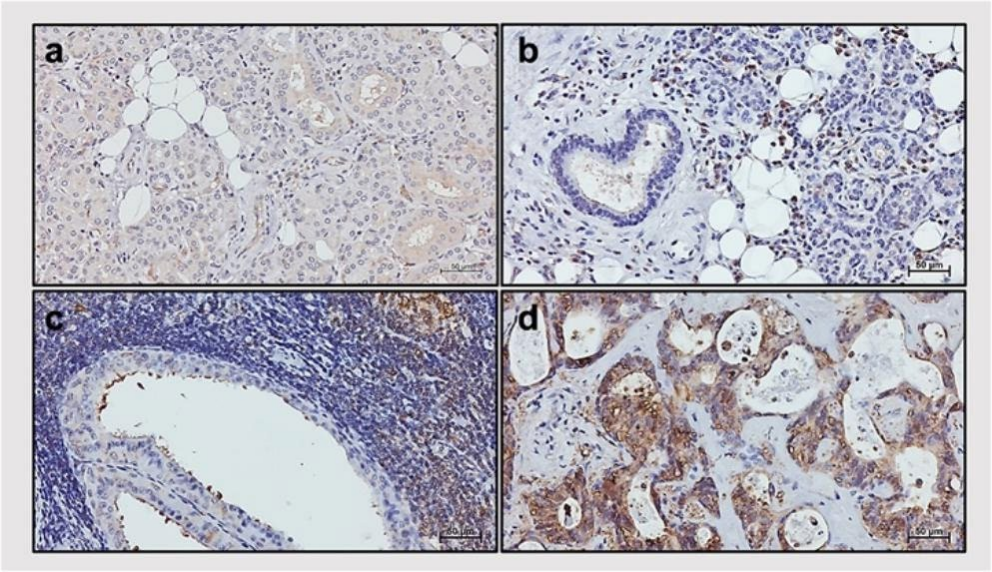
Immunohistochemistry of Hsp90 in salivary gland tissue. (a) normal salivary glands, (b) sialadenitis, (c) Warthin’s tumor, and (d) mucoepidermoid carcinoma. The chaperone appears stained brown, with differences between the specimens in quantity and distribution. Magnification x200

As part of our current ongoing study, we sought to investigate the role of different molecular chaperones in normal and tumorous tissue of the major salivary glands. Initially, we focused on two chaperones, namely Hsp27 and Hsp60, and assessed their tissue levels in human submandibular glands, using two different techniques, Immunohistochemistry (IHC) and Immunofluorescence (IF) (Basset et al. 2021). Hsp27 and Hsp60 showed a significant decrease in pleomorphic adenoma (benign) and adenoid cystic carcinoma (malignant) when compared with normal counterparts. (Basset et al. 2021). Work currently performed in our laboratory on tissue levels of Hsp90 in tumors of the submandibular and parotid glands is revealing interesting differences between pathologies. Preliminary observations indicate that Hsp90 is present at different levels in normal healthy glands (Fig. 2a), sialadenitis (Fig. 2b), Warthin’s tumor (Fig. 2c), and mucoepidermoid carcinoma (Fig. 2d). Thus, our data are encouraging at this time in as much as they indicated that tissue levels of Hsp90 deserve further investigation to determine their value as biomarkers useful in differential diagnosis and disease monitoring in tumors of the salivary glands.

**Fig. 3:**
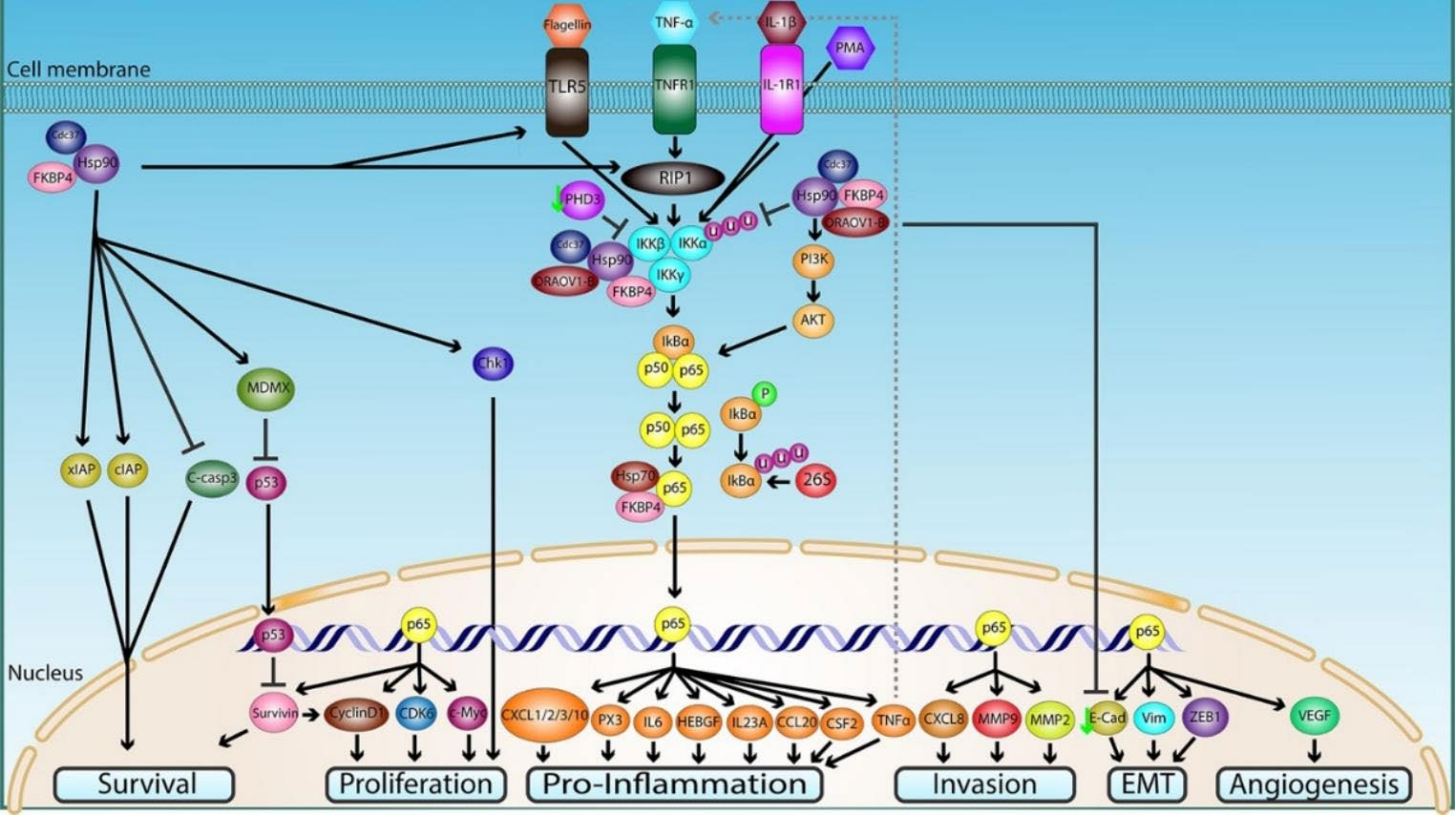
Hsp90 molecular mechanisms and regulation of the NF-kB signaling pathway in cancer. Constitutive or inducible (flagellin, TNF-α, IL-1β, and phorbol ester (PMA)-activated) NF-kB pathway is initiated by activation of the IKK complex. Hsp90 with its co-chaperones Cdc37 and FKBP4 and oncogene ORAOV1-B bind and stabilize the complex by abrogating IKKα and IKKβ degradation by the ubiquitin-proteasome system. Hsp90 also modulates the kinase activity of IKKα and IKKβ. PHD3 is downregulated in cancer cells to impede its inhibitory effect on Hsp90 and IKKβ interaction (green arrow on the left of the PHD3 icon). In the TNF-α activated NF-kB (otherwise referred to as p50/p65 heterodimer) pathway, Hsp90 regulates the activity of RIP1 to stimulate IKK activation. In flagellin-activated NF-kB, Hsp90 plays a role in upregulating the expression of TLR5, the cell-surface receptor for flagellin. Upon activation of IKKβ, IkBα is phosphorylated and degraded by the 26 S proteasome, releasing p65 and allowing its translocation into the nucleus, with the aid of Hsp70/FKBP4 complex, to bind to the promoter region of specific regulatory genes, including: (1) the cell-cycle and proliferation genes survivin, cyclin D1, CDK6, and c-myc; (2) the pro-inflammatory genes CXCL1, CXCL2, CXCL3, CXCL10, PX3, IL6, HEBGF, IL23A, CCL20, CSF2, and TNFα; (3) the migration and invasion genes MMP2, MMP9, and CXCL8; (4) the EMT genes E-cadherin (E-Cad), vimentin (Vim), and ZEB1); and (5) the angiogenesis gene VEGF. E-cadherin expression is repressed by ORAVO1-B (green arrow on the left of the E-Cad icon). TNF-α is involved in a positive feedback loop (dotted line) that potentiates the activation of the TNF-α-NF-kB pathway. Hsp90 also regulates the activation of Akt that in turn activates the NF-kB pathway. Hsp90 stabilizes MDMX that suppresses p53 from inhibiting survivin. NF-kB activation together with p53 inhibition up-regulate survivin expression promoting cell survival. Survivin expression enhances cyclin D1 activation thus mediating proliferation of cancer cells. Chk1 activity is potentiated by Hsp90 to promote proliferation. Hsp90 modulates apoptosis in cancer cells not only by its actions on survivin, but by upregulating xIAP, and cIAP and by inhibiting cleaved caspase 3 (C-casp3). All these processes, together, render the tumor cell prone to growth, invasion of adjacent tissues, and metastasization

Quantification of tissue Hsp90 at early stages of carcinogenesis to assess the risk of progression and the overall survival may be a promising endeavor in what concerns diagnosis, prognostication, and decision-making regarding treatment. Furthermore, quantification of Hsp90 (and other components of the CS) in tissues and mapping their distribution in them to identify changes in tumor cells and tissues, will provide information useful for understanding the molecular mechanisms of carcinogenesis. The data may also reveal points of attack with anti-cancer drugs. Here, we will summarize some of the molecular mechanisms in which Hsp90 has been shown to play a role in carcinogenesis, involving the PI3K/AKT/NF-kB pathway.

NF-kB is a transcription factor and a pleiotropic activator of several cellular processes involved in cancer, including cellular proliferation, cell cycle, inflammation, apoptosis, and migration (see for example Yeramian et al. 2016). The pathway starts with activation of the TNF-α receptor 1 (TNFR1) by TNF-α that causes conformational changes and subsequent detachment of the silencer of death domain (SODD) from the intracellular death domain of TNFR1. After the release of SODD, tumor necrosis factor receptor type 1-associated death domain protein (TRADD) is recruited to the death domain of TNFR1. TRADD then recruits another protein, TNF receptor-associated factor 2 (TRAF-2), that binds to TRADD. TRADD/TRAF-2 complex recruits IAP (inhibitor of apoptosis protein) to inhibit the TNF-α mediated apoptosis pathway and to allow initiation of NF-kB pathway. TRAF-2 will recruit RIP1/RIPK (receptor-interacting protein kinase). RIPK will activate IKK (IkB kinase) by phosphorylation. In normal physiological conditions, NF-kB is bound by IkBα, which diminishes its transcriptional activity by masking its nuclear localization signal (NLS). IKKβ phosphorylates IkBα and drives its proteasomal degradation and subsequent release of the NF-kB protein that translocates to the nucleus (Brown et al. 2008; Bakkar and Guttridge 2010; Xia et al. 2018). The NF-kB pathway can be activated through cross talk with PI3K/AKT, highlighting the complexity and the synergism between those two distinct pathways (Yeramian et al. 2016). Hsp90 promotes carcinogenesis by potentiating the NF-kB survival pathway through activation of Akt and inhibiting apoptotic pathways by blockade of caspase 3 cleavage and up-regulation of xIAP (x inhibitor of apoptosis protein) and cIAP (Kim et al. 2016; Qin et al. 2017). Hsp90 with its co-chaperone Cdc37 are required for the formation of active AKT and IKK complex that phosphorylate and degrade IkB to allow NF-kB nuclear translocation and transcriptional activity (Chen et al. 2002). Hsp90 modulates tumor cell proliferation and migration via activation of the PI3K/AKT and MAPK/ERK signaling pathways in endometrial carcinoma (EC) (Yeramian et al. 2016). Hsp90 enhances NF-kB activity in EC by regulating IKKα and IKKβ in EC under normal and hypoxic conditions (Yeramian et al. 2016). Inhibition of Hsp90 has been shown to be more effective in abrogating NF-kB transcriptional activity than IKKβ-specific inhibitors, thus highlighting the complexity of the network signaling in neoplastic cells and demonstrating the advantage of adopting a compound with pleiotropic targeting pathways rather than single pathway inhibitors (Nottingham et al. 2014). While Hsp90 inhibition abolishes both IKKα and IKKβ protein expression by suppressing Hsp90 protection from proteasomal degradation, specific IKKβ inhibition triggers compensatory IKKα activation of the classical NF-kB pathway (Lam et al. 2008). NF-kB signaling pathway is often deregulated and its expression is increased in most cancers as it promotes proliferation via increased activation of cytokines and pro-inflammatory genes (Karin and Greten 2005; Xia et al. 2018). Hsp90 is required for the folding and maturation of nascent IKKα and IKKβ in cancer (Broemer et al. 2004). Hsp90 binds to the IKK complex, stabilizes, and modulates the activity of IKKα and IKKβ and impedes polyubiquitination-mediated 26 S proteasomal degradation (Broemer et al. 2004). Hsp90 is believed to be essential for the activation of the constitutive and inducible NF-kB pathways (Broemer et al. 2004). For the TNF-α activated NF-kB pathway, Hsp90 regulates the activity of RIP1 and IKKβ (Broemer et al. 2004; Qu et al. 2013). FKBP4 is a member of the immunophilin family and a co-chaperone of Hsp90 the expression of which is increased in breast, prostate, hepatocellular, and lung cancers with implications of poor prognosis (Ward et al. 1999; Lin et al. 2007; Liu et al. 2010; Lacombe et al. 2013; Joshi et al. 2017; Zong et al. 2021). FKBP4 potentiates NF-kB pathway and transcriptional activity through IKKβ and enhances phosphorylation of IKKα/β, IkB and RelA (p65) in carcinoma (Zong et al. 2021). FKBP4 PPI domain interacts with IKKγ and is necessary for interaction between IKK and Hsp90; FKBP4 may also regulate the levels of downstream targets of NF-kB such as MMP9, c-myc, cyclin D1 and CDK6; and FKBP4 associates with Hsp70 and forms a complex that favors p65 translocation into the nucleus (Zong et al. 2021). 1-B (ORAOV1-B), a long noncoding RNA (lncRNA), which is a splice variant of the oncogene ORAOV1, was found overexpressed in oral cancer (Luo et al. 2021). ORAOV-1B binds to the Hsp90/RIP1/IKK complex to activate the TNF-α-NF-kB pathway and its overexpression may also stimulate the expression of migration and invasion proteins MMP2 and MMP9 (Luo et al. 2021). Prolyl hydroxylase 3 (PHD3) expression is decreased in colorectal cancer (Xue et al. 2010). PHD3 disrupts the interaction between IKKβ and Hsp90 and hinders the TNF-α activated NF-kB pathway. Specifically, PHD3 inhibits the TNF-α induced phosphorylation of IKKβ and competes with HSP90 for binding at IKKβ (1-307) and IKKβ (308–580) domains (Xue et al. 2010). The TNF-α/NF-kB activated pathway is associated with epithelial mesenchymal transition (EMT) and metastasis as it upregulates the mesenchymal markers vimentin and ZEB1 while downregulating the epithelial marker E-cadherin and may activate genes involved in inflammation and invasion such as IL6, CXCL1, HEBGF, PTX3, CXCL2, CXCL3, CXCL10, CSF2, CCL20, IL23A; all of which are downstream targets of NF-kB (Nottingham et al. 2014). Interestingly, the TNF-α/NF-kB pathway represents a positive feedback loop as it actively transcribes TNFα gene, which in turn re-activates and enhances the pathway and promotes metastasis (Luo et al. 2021). Hsp90 may be the main chaperone responsible for the folding and maturation of TLR5 surface receptors in flagellin-TLR5 activated NF-kB pathway in carcinogenesis (Na et al. 2018).

Hsp90 promotes survival, cell proliferation, and anti-apoptosis in hepatocellular carcinoma by down-regulating tumor-suppressor protein p53 (p53) and simultaneously up-regulating the survival proteins NF-kB, survivin, and cyclin D1 (Leng et al. 2012). Hsp90 forms a complex with survivin (strong inhibitor of apoptosis) and stabilizes its mature form through physical binding, which results in subsequent inhibition of apoptosis (Fortugno et al. 2003; Siegelin et al. 2009). p53 transcriptional function and apoptotic activity is repressed by MDM2 (murine double minute-2) and MDMX (murine-double minute-X) proteins; the latter stabilized by Hsp90 (Goldstein et al. 2011; Vaseva et al. 2011; Kim et al. 2016). The survivin’s promoter region contains NF-kB and p53 binding sites that regulate its expression (Otaki et al. 2000; Sah et al. 2006). Survivin is expressed during the G2/M phase of the cell cycle and its expression can affect cyclin D1 expression and thus cell proliferation (Ambrosini et al. 1997; Ai et al. 2006). Cyclin D1 is also a target gene of NF-kB and its expression is downregulated upon Hsp90 inhibition in carcinoma (Hartman et al. 2020). Activation of Chk1 may also require Hsp90 and may promote proliferation in carcinoma (Kim et al. 2016). Hsp90 may also mediate angiogenesis in cancer by upregulating both the transcript and protein levels of VEGF (Hartman et al. 2020). While isoflavone-deprived soy peptide is not considered a specific inhibitor of Hsp90, it inhibited Hsp90 and NF-kB expression and suppressed mammary tumorigenesis by inducing apoptosis (Park et al. 2009). It is worth mentioning that isoflavone-deprived soy peptide had a similar inhibitory effect to that of Hsp90-specific inhibitors as it downregulated VEGF and c-casp3 while it upregulated p21 and p53 expression (Park et al. 2009). Hsp90 upregulates of the MMP9 and CXCL8 genes, thus increasing protein levels of MMP9 and CXCL8 that participate in migration and invasion in neoplastic cells (Hartman et al. 2020; Zong et al. 2021). Based on the published data, we propose a model depicting the mechanisms by which Hsp90 interacts with the various NF-kB components and thus promotes tumor development, growth, and metastasization (Fig. 3).

## Hsp90 in cancer treatment

### Natural compounds and first-generation inhibitors

Progress in the understanding the Hsp90 structure, dynamics, and mechanisms of action, including its ATPase activity led to the discovery of compounds that competitively bind to the N-terminal-ATP binding domain and disrupt its function. In this sub-Section, we will concentrate on the N-terminal Hsp90 inhibitors and their limitations. The naturally occurring Hsp90 inhibitor geldanamycin (GA), a 1,4-benzoquinone ansamycin antibiotic derived from *Streptomyces hygroscopicus*, was discovered in the early 1990s (Huryn and Wipf 2013). GA showed potency in killing cancer cells, however never made it into clinical trials due to its in vivo toxicity, instability, and poor solubility (Biamonte et al. 2010). GA-dependent hepatotoxicity is mainly due to the GA’s reactive quinone moiety that produces superoxide radicals, resulting in cell death independent of Hsp90 inhibition (Samuni et al. 2010). Radiciol (RC), isolated from the fungus *Monosporium bonorden*, is another naturally occurring Hsp90 inhibitor (Huryn and Wipf 2013). GA and RC were never selected for clinical trials due to their adverse effects (AE). However, their anti-cancer effect was the driving factor that led to designing semi-synthetic derivatives that would be more efficient and that, later on, were used for clinical studies. The C-17 position of GA was responsible for its mechanism of action and liver toxicity, thus new compounds were synthesized in which a substituent was added to the C-17 position to revert the toxicity of GA (Gorska et al. 2011). These semi-synthetic drugs are known as first generation Hsp90 inhibitors and include: (1) 17-N-allylamino17-dimethoxygeldanamycin (17-AAG; tanespi-mycin); (2) 17-AAGH2 (IPI-504; retaspimycin); (3) 17-AG (IPI-493); and (4) 17dimethylamino-17-dimthoxygeldanamycin (17-DMAG; alvespimycin) (Sanchez et al. 2019). Most of these drugs entered Phase I clinical trials but none of them made it past Phase II (Sanchez et al. 2019).

### Second-generation inhibitors

While first generation Hsp90 inhibitors showed great potential pre-clinically, they still exhibited limitations in clinical studies that impeded their progress into Phase III clinical trials. Thus, efforts were put into improving binding affinity, potency, AE, and bioavailability. Fully synthetic small molecules were designed as novel inhibitors of Hsp90. They are known as second generation of Hsp90 inhibitors and include: (1) Purine-based inhibitors (PU-3, BIIB021, BIIB028, MPC-3100, PU-H71, and Debio0932); (2) Benzamide inhibitors (SNX-5422); (3) Resorcinol containing inhibitors (AUY922, STA9090, AT13387 and KW-2478); and (4) miscellaneous inhibitors (XL888, HSP990, DS2248) (Sanchez et al. 2019).

Ganetespib is currently the most advanced and potent second-generation inhibitor of Hsp90 and the most studied clinically, as by 2021, the drug STA-9090 has officially entered a total of 38 (Phase I to Phase III) clinical trials (Ray-Coquard et al. 2019; Sanchez et al. 2019). While second-generation Hsp90 inhibitors circumvented liver toxicities, ocular toxicities manifested in some of the drugs such as AUY922 in the form of night blindness, blurred vision, and visual disturbances (Pillai and Ramalingam 2018). This is mainly linked to apoptosis in the outer layer of the retina. No ocular toxicity was reported with Ganetespib as it did not elicit apoptosis although it produced diarrhea and fatigue as the main AE (Ray-Coquard et al. 2019).

The main concern and major limiting factor that prevented FDA approval for N-terminal domain Hsp90 inhibitors is their dosage limitations. This could be explained by the pan-inhibitory effect of the semi-synthetic and fully synthetic compounds that trigger the HSR and increase Hsp70 expression as a pro-survival response, which brings the need of higher doses to maintain the inhibitory effect, but this is paralleled by an increase in toxicity (Wang et al. 2019; Sanchez et al. 2019).

### Isoform-specific inhibitors

Since pan-inhibition back-fired with the regulatory feedback loops in the form of HSR, other more efficient Hsp90-dependent anticancer therapies were investigated, focusing on inhibition specific isoforms. Pimitespib (or TAS-116) is an example of a novel selective drug that targets cytosolic Hsp90α and Hsp90β isoforms and has recently entered a phase III clinical trial (Honma et al. 2021). TAS-116 shows great antitumor potential as it improved progression-free survival and overall survival with tolerable and manageable AE (Honma et al. 2021). More advanced and specific inhibitors for specific isoforms have been designed; however they have not entered clinical trials yet (Sanchez et al. 2019). Inhibiting solely Hsp90β seems to be efficient in suppressing HSR by degradation of HSF1 (Blair et al. 2019). The possibility of inhibiting Hsp90 without the drawback of the HSR should be explored exhaustively because it offers a potentially efficient way for cancer treatment.

### C-terminal and co-chaperone inhibitors

Another novel approach to target Hsp90 in cancer while circumventing HSR and HSF1/Hsp70 upregulation is by disrupting the interaction between the chaperone and the co-chaperone containing TPR domain on the CTD. Novobiocin and its analogue KU-174 are the most prominent examples. Their binding on to the CTD motif leads to aberrant chaperone function and ubiquitination-mediated degradation of the client protein (Yun et al. 2004; Donnelly and Blagg 2008).

DDO-5936 is newly discovered and synthesized small molecule that targets Hsp90-Cdc37 and leads to the disruption of the protein-protein interaction (Wang et al. 2019, 2020). Cdc37 is a co-chaperone of Hsp90 involved in binding to unmature kinases and aiding their proper folding and maturation. Kinases are known to be heavily involved in carcinogenesis. DDO-5936 demonstrated anti-proliferative effects with cell cycle arrest at G0/G1 (Wang et al. 2019, 2020). Other Hsp90 non-oncogenic protein clients were not affected by DDO-5936 in contrast with the pan-inhibitory effect of first and second-generation inhibitors. Moreover, DDO-5936 has a specific independent binding site on Hsp90 and does not affect the binding of other co-chaperones or block the ATPase activity on NTD, thus maintaining the normal activity of the chaperone and avoiding unwanted toxicity (Wang et al. 2019, 2020).

The data in general indicate that while most Hsp90 single-action inhibitors show anticancer potential, a combination therapy will probably be more efficacious. This is not surprising because Hsp90 is a member of the CS and this is a physiological system whose members are functionally interrelated throughout the body and thus, what happens to one member may affect others and trigger compensatory pathways within the CS. Therefore, manipulation of Hsp90 is bound to produce the expected effect, e.g., inhibition, as well one or more unexpected effects, some of which might very well compensate for the function inhibited.

## Conclusion

The CS is a physiological system distributed throughout the body, but its various components are present in different concentrations and assemblages that are distinctive of cell and tissue type. We have been studying the CS in the salivary glands, important organs for the physiological wellbeing of the entire organism but somewhat ignored by clinicians and pathologists in comparison with other anatomical entities such as, for example, the liver, heart, and various others. Consequently, knowledge of the CS in the salivary glands is only now beginning to progress, which is key to advancing our understanding of the glands’ development, functioning under normal conditions and in response to stressors, and diseases, including cancer. Thus far, we and few others have studied some of the main components of the CS in salivary glands, namely molecular chaperones. These are the chief members of the CS and play a critical role in the maintenance of protein homeostasis as well as in other cellular processes pertaining to immunity, inflammation, and carcinogenesis. Chaperones are typically cytoprotective, but if abnormal they can cause diseases, the chaperonopathies. Little is known on salivary gland chaperonopathies, and we are conducting studies on them, focusing on cancer. We have established that some molecular chaperones display quantitative and distribution patterns that are typical of certain malignancies and, therefore, are promising biomarkers for use in differential diagnosis, prognostication, and patient management. In addition, the data provide clues on how to develop anti-cancer therapies centered on chaperones, namely chaperonotherapy. Some chaperones are known to favor carcinogenesis under certain circumstances and one of them is Hsp90. We are in the process of mapping and quantifying this chaperone in normal human salivary glands and in their tumors. The immediate goal is to assess the value of quantification and mapping of Hsp90 for differential diagnosis between tumor and non-tumoral pathologies, and between tumor types. The long-term aim is to obtain information that could help in the development of chaperonotherapy targeting Hsp90 for tumors of the salivary glands. In this review, we summarize the structure, physiology, and participation of Hsp90 in carcinogenesis, as well as the strategies and methods tried thus far to treat cancer targeting this chaperone. It becomes clear that Hsp90 has been a hot topic in the cancer research field for the last two decades. The importance of this chaperone resides in the identity of its clients, which are proteins critical for the survival, growth, and dissemination of tumor cells, and which need the chaperone for their folding and maturation pathways, for example oncogenic mutated proteins and kinases. We discuss Hsp90 molecular mechanisms that are involved in carcinogenesis. For instance, we consider how Hsp90 stabilizes and modulates the IKK complex and the PI3K-Akt kinase axis to regulate NF-kB transcription activity, inducing different hallmark cancer responses (pro-inflammation, proliferation, survival, angiogenesis, invasion, and EMT). We also highlight the sequential discoveries of Hsp90 inhibitors used for negative chaperonotherapy, namely the inhibition of the chaperone, which in some instances showed antitumor activity, but the side effects were considerable. More research is underway to develop inhibitors, and combinations thereof, with less damaging side effects. The road is difficult to negotiate because of the very nature of the CS, in which there is extensive cross talk and “help” between members and built-in backup pathways to replace damaged ones, such as those inhibited by medication.
